# microRNA evaluation of unknown primary lesions in the head and neck

**DOI:** 10.1186/1476-4598-8-127

**Published:** 2009-12-23

**Authors:** Emma V Barker, Nilva K Cervigne, Patricia P Reis, Rashmi S Goswami, Wei Xu, Ilan Weinreb, Jonathan C Irish, Suzanne Kamel-Reid

**Affiliations:** 1 Department of Head and Neck Surgical Oncology, Princess Margaret Hospital, Toronto, Ontario M5G 2M9, Canada; 2 Department of Applied Molecular Oncology, Princess Margaret Hospital, Ontario Cancer Institute and University Health Network, Toronto, Ontario M5G 2M9, Canada; 3 Department of Genetics, Biosciences Institute, UNESP, Botucatu, SP, Brazil; 4 Department of Laboratory Medicine and Pathobiology, University of Toronto, Toronto, Ontario M5S 1A8, Canada; 5 Department of Biostatistics, Princess Margaret Hospital, Toronto, Ontario M5G 2M9, Canada; 6 Department of Pathology, Toronto General Hospital, University Health Network, Toronto, Ontario M5G 2M9, Canada

## Abstract

Unknown primary malignancy in the head and neck is not an infrequent diagnosis for patients with metastatic cervical lymph nodes. Although linked with a relatively good prognosis following radiation treatment, widespread radiation is coupled with significant morbidity. Altered microRNA (miRNA) expression has been associated with both cancer progression and metastasis. We sought to determine whether miRNA expression analysis could be used as a diagnostic tool to discover the primary site of malignancy, within the head and neck. We used quantitative real-time PCR to identify miRNA expression profiles of squamous cell carcinoma of the tonsil, base of tongue and post-nasal space, as well as their corresponding metastatic lymph nodes, from 6 patients. Our results revealed that each cancer maintained its expression profile between the primary site and the nodal metastasis (r = 0.82, p < 0.0001). In addition, each anatomical sub-site maintained a distinct miRNA profile between individual patients (r = 0.79, p < 0.0001). Finally, between sub-sites, the miRNA profiles were distinct (p < 0.0001). As proof of principle, our study provides an indication that miRNA expression analysis may be useful to compare the primary lesion and local metastatic disease. This may be clinically relevant to predict the primary site of origin of metastatic disease, when the primary site remains obscure.

## Findings

Cancer of unknown primary site is defined as a histologically confirmed metastasis in the absence of an identifiable primary tumour, despite a standardized diagnostic approach [1]. Within the head and neck, up to 10% of metastatic squamous cell carcinoma has an unknown primary site, despite extensive diagnostic investigations [2]. Positron emission tomography (PET) scanning has helped reduce this number [3]. Patients with a known primary site have a better prognosis [2]. This may be because if the origin of the disease is known, specific therapeutic regimes can be used, including reduced radiation fields, thereby minimizing therapy associated morbidity. In contrast, a widespread radiation field used in unknown primary disease is associated with increased morbidity.

Patients presenting with metastatic disease within their cervical lymph nodes frequently have their primary disease within one of three sites; the tonsil, the base of tongue (BOT) or the post nasal space (PNS). However, despite a thorough clinical history and examination, including biopsies, the primary site of disease may remain concealed.

microRNA (miRNA) are approximately 22 nucleotides, non-coding RNA molecules that bind to complementary messenger RNA (mRNA) and regulate post-transcriptional gene expression by either degrading mRNA or inhibiting its translation. It has been estimated that the number of miRNAs present in the human genome is approximately 1000 [4]; to date, 677 were identified (miRBase, The Sanger Institute) and they are predicted to regulate at least 30% of mRNA transcripts [5,6]. miRNA function in concert with tumor suppressors and oncoproteins to regulate key cellular pathways involved in development, differentiation and tumorigenesis [7-9]. miRNA expression profiling of human tumors has identified signatures associated with diagnosis, staging, progression, prognosis, and response to treatment [6,10]. Importantly, miRNAs also appear to be a molecular-fingerprint that may help predict the primary site of metastatic disease [11].

In this study, we investigated miRNA expression from primary cancer (tonsil, BOT and PNS) and their corresponding metastatic cervical lymph nodes to determine the similarity of miRNA profiles between the primary and metastatic lesions. Such miRNA profiles may be clinically useful to help predict the primary site of disease.

This study was approved by the University Health Network Research Ethics Board (07-0511-TE). The cancer registry was used to identify patients with biopsy proven head and neck squamous cell carcinoma (HNSCC) with regional cervical lymphadenopathy. Formalin-fixed paraffin-embedded (FFPE) specimens from both the primary site (tonsil, base of tongue and post-nasal space) and corresponding metastatic lymph node were sought from Toronto General Hospital, Canada. We collected 12 tissue samples from 6 patients (2 samples/patient) for the training set. An additional 6 patients (N = 12 samples) were collected for validation analysis. Clinical details are described in Table 1.

**Table 1:** Patient clinical data.

Patient	Age	Sex	Tobacco	Alcohol	Primary Tumor Site	Tumor Grade	Stage	Outcome	Last FU
*Training set*									

1	55	F	Y	Y	Tonsil	PD	T1N1	AWD	05/2004

2	44	F	N/A	N/A	Tonsil	MD	T1N2	ANED	05/2001

3	73	M	Former	Y*	BOT	PD	T1N2	ANED	03/2000

4	56	M	N	Y*	BOT	MD	T1N2	ANED	11/2009

5	44	F	N	N	PNS	NPC	T4N1	ANED	04/2009

6	41	M	N	Y*	PNS	NPC	T2N2	ANED	08/2009

*Validation set*									

7	61	M	Y	Y	Tonsil	MD	T2N2b	ANED	09/2009

8	63	M	Y	Y	Tonsil	MD	T2N0	ANED	05/2009

9	58	M	N	Y*	BOT	PD	T1N2b	ANED	10/2009

10	47	F	N	N	BOT	MD	TxN3	ANED	08/2009

11	57	M	Y	N	PNS	NPC	T1N2	ANED	07/2009

12	56	M	Y	N/A	PNS	NPC	T1N3	ANED	05/2009

Former: patient quit smoking in 1984; Alcohol; Y*: occasional drinker

BOT: base of tongue; PNS: post-nasal space

PD: poorly differentiated; MD: moderately differentiated

AWD: Alive with disease; ANED: Alive with no evidence of disease

FU: Follow-up (Month/Year)

None of the patients have had previous irradiation.

Histopathological analysis of all tissues was performed by a head and neck pathologist (IW) to ensure the presence of carcinoma in at least 80% of each tissue section. Total RNA was isolated and purified from all samples using the RecoverAll™ Total Nucleic Acid Isolation Kit (Ambion, Austin, TX, USA), according to the manufacturer's protocol. Samples were stored at -80°C and RNA quality and quantity were evaluated by spectrophotometry.

We used the TaqMan Low Density Array (TLDA) Human MicroRNA Panel and Multiplex RT Pools of eight 48-miRNA sets (Applied Biosystems, Streetsville, ON, Canada) for global miRNA expression analysis by quantitative real-time PCR (QRT-PCR). Briefly, total RNA was converted into specific cDNA derived from mature miRNAs, and then quantified using the TaqMan miRNA assay by QRT-PCR. The TLDA card contained 365 lyophilized human miRNA sequences, plus three small nucleolar RNAs (snoRNAs), *RNU6B, RNU48 *and *RNU44*, used as endogenous controls. Data was quantified and analyzed using the Sequence Detection System software (v. 2.3) (Applied Biosystems). The relative expression of each miRNA was normalized against endogenous controls using the equation 2^-ΔCt^, where ΔCt = (Ct_miRNA_-Ct_snoRNAs_). miRNA expression levels in primary tumour and lymph nodes were calculated relative to a commercially available Universal Reference RNA (Stratagene).

**Figure 1:**
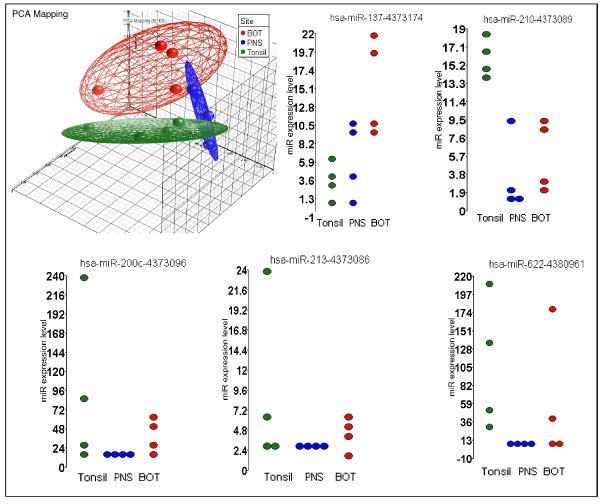
**Principal component analysis based on the differential expression levels of 5 miRs (miR-137, miR-210, miR-200c, miR-213, and miR-622)**. These miRs showed the most significant differential expression according to each tumor site. The dot plots represent the relative expression levels of each miR across all sites.

The pair-wise correlation coefficient of miRNA expression between the primary tumor and corresponding lymph node was significant for each patient (0.82, p < 0.0001), indicating that miRNA expression remained consistent between the primary lesion and local metastatic disease. In addition, the correlation coefficient within the same site, but between different patients, was 0.76 (p < 0.0001) for the tonsil, 0.83 (p < 0.00001) for the BOT and 0.90 (p < 0.0001) for the PNS. Overall, the similarity between patients in each sub-site was 0.79 (p < 0.0001). These data were subjected to Principal Component Analysis (PCA) using Partek^® ^Genomics Suite software, version 6.4 Copyright^© ^2009, Partek Inc., St Louis, MO, USA [12]. Figure 1 shows the PCA and expression values for 5 representative miRNAs, which were significantly differentially expressed across samples.

**Figure 2:**
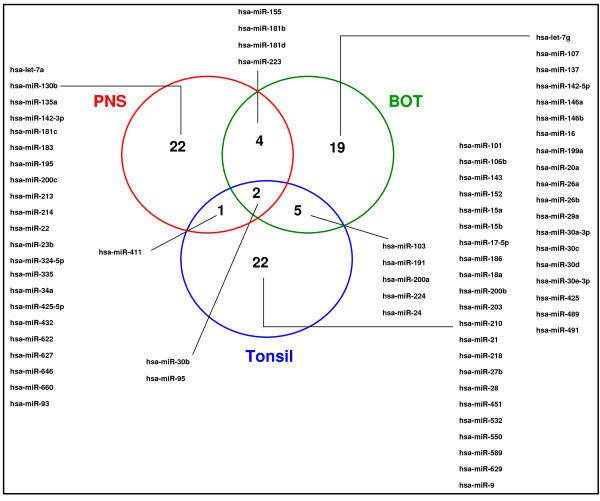
**Venn diagram showing the top most similar miRNAs within each anatomical sub-site (BOT: base of tongue, PNS: post nasal space)**.

We next identified the top miRNA expression values with the greatest similarity within each sub-site, totaling 75 miRNAs, using residual sum of squares (RSS: 0.0008-0.01). A total of 12 out of 75 miRNAs were common between any 2 sub-sites combined. Excluding these 12 overlapping miRNAs, a total of 63 were then used to compare the miRNA expression profile between the three primary sites (tonsil, BOT and PNS) (figure 2). The mixed model regression analysis showed a significant difference between miRNA profiles from tonsil, BOT and PNS (p < 0.0001), suggesting that discrete anatomical locations within the head and neck expressed distinct miRNA profiles. The pair-wise difference between tonsil and PNS was p < 0.0001; tonsil and BOT was p = 0.14; and PNS and BOT was p = 0.0006.

**Figure 3:**
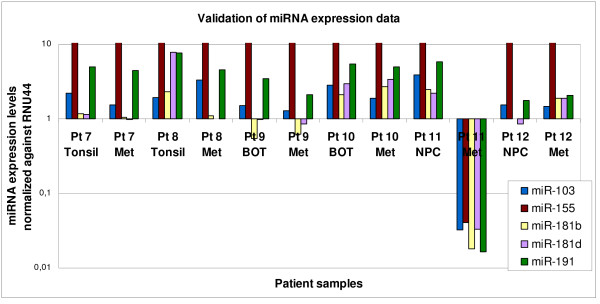
**QRT-PCR validation data, showing the relative expression of 5 miRs (miR-103, miR-155, miR-181b, miR-181d and miR-191), similarly expressed across all subsites in the training set**. miR expression is normalized against RNU44. Expression levels are log transformed (log_10_); values above 1 indicate over-expression; values below 1 indicate under-expression.

We performed QRT-PCR validation analysis, as previously reported by our group [13]. We selected 5 miRNAs for validation (miR-103, miR-155, miR-181b, miR-181d, and miR-191); these miRNAs were selected based on their similar expression levels in primary tumor and metastatic tissues, among the distinct subsites. RNU44 was used as endogenous control. Gene expression values were calculated using the ΔΔCt method. We confirmed that miR-155 was highly expressed in all samples, regardless of tumor site (Median expression level = 36.9) relative to Human Universal RNA (expression = 1); miR-181b and miR-181d were similarly expressed (Median expression = 1.1, for both). Increased expression levels were confirmed for miR-103 (Median = 1.4) and miR-191 (Median = 4.4), as compared to the Universal RNA (figure 3). Interestingly, one lymph node metastasis sample (Pt. 11, NPC) showed under-expression of all these 5 miRNAs, suggesting the need for further examining the biological significance of deregulated miRNA expression in primary tumours and their metastases.

Overall, miRNA expression patterns may be site specific within the head and neck (e.g, tongue *vs*. nasopharynx). The embryological events that result in the formation of the head and neck may be the reason why distinct anatomical sites contain dissimilar miRNA profiles. In brief, the posterior tongue arises from the third pair of branchial or pharyngeal arches. In contrast, the tonsil originates from the second pouch endoderm (forming the surface epithelium and the lining of the crypts of the palatine tonsil) and the mesenchyme around the crypts differentiates into lymphoid tissue that organizes into the lymphatic nodule of the palatine tonsil. The nasopharynx is more indistinct and is derived from both the first and second arches. It has been shown that different tumour types have distinct patterns of miRNA expression and this profile reflects the developmental origin of the tissue [11]. In addition, within a single developmental cell lineage, distinct patterns of miRNA expression can also be observed, representing different mechanisms of transformation such as chemical or viral carcinogens [11]. Lu *et al *[11] used miRNA expression to define non-head and neck, poorly differentiated tumours when the histology was not diagnostic. 12/17 patients were diagnosed correctly in contrast to 1/17 when a mRNA-based classifier was used. Another advantage of a miRNA centered diagnostic tool is that, in contrast to mRNA, miRNA is not degraded in formalin-fixed, paraffin-embedded clinical tissues. In addition, miRNA may be important for both diagnostic and prognostic purposes: miRNA over-expression was found associated with oral carcinoma progression [13] and changes in miRNA levels were correlated with survival in lung carcinoma [14].

miRNAs are known to be important in HNSCC [15-17] including tongue [18,19] and nasopharyngeal [20] carcinomas. hsa-miR-205 has been shown to be highly expressed in HNSCC cell lines but not in lung, breast, colorectal, prostate or pancreatic cell lines [16]. In addition, hsa-miR-205 was over-expressed in tonsillar carcinoma compared with normal tonsil (unpublished data from same authors). In our analysis, has-miR-205 showed very high expression levels in both primary tumour and metastatic disease from all subsites. Interestingly, hsa-miR-205 over-expression was recently shown to be specific for detection of metastatic disease in head and neck carcinoma [21]. In contrast with our results, Tran *et al *[16] showed an absence of site-specific miRNA expression profile between the tongue, oropharyngeal and laryngeal cancer cell lines. This difference could be attributed to the fact that potentially, cell lines express different miRNA expression profiles than either normal tissue or an associated tumour.

We were interested to determine if miRNA expression could afford a diagnostic advantage. In thyroid malignancy, miRNA expression analysis is currently being investigated as a diagnostic tool [22-25]. Specifically, miR-146b is over-expressed in papillary carcinoma, but benign pathology reveals a lower expression for this miRNA [24]. In this study, miRNA was successfully extracted from fine needle aspirates (FNA).

Ultimately, in the unknown primary setting, we would propose to aspirate cells (FNA) from the neck node and use cells for miRNA analysis. Over time, a bank of expression profiles from both normal tissue (from healthy subjects) and carcinoma from each sub-site would be formed and used to help determine the most likely primary site of disease.

In summary, this is the first study to evaluate the use of miRNA as a diagnostic aid to predict the origin of an unknown primary tumour in the head and neck. We showed that the miRNA expression profile between the primary lesion and local metastatic disease remains consistent for an individual patient and between different patients within an individual site. In addition, the analysis of a subgroup of similarly expressed miRNAs within each site showed that each primary site has a distinct miRNA expression profile. In the future, miRNA expression profiling analysis may help predict the site of origin of the metastatic disease, when the primary site remains clinically obscure. This will potentially lead to improved patient management and decreased morbidity.

## Abbreviations

miRNA: microRNA; HNSCC: Head and neck squamous cell carcinoma; BOT: Base of tongue; PNS: Post nasal space; FFPE: Formalin-fixed paraffin-embedded; snoRNAs: Small nucleolar RNAs; RSS: Residual sum of squares.

## Competing interests

The authors declare that they have no competing interests.

## Authors' contributions

EVB designed the study, identified patients and drafted the manuscript. NKC ran the TLDA assays, performed data analysis and helped writing the manuscript. PPR helped with the study design, data analysis and validation, and performed editorial revision of the manuscript. RSG helped identify patient samples, performed validation and data analyses and helped with editorial revision of the manuscript. WX performed statistical analyses. IW identified the patients (pathology) and helped with editorial revision of the manuscript. JCI helped with the study design and gave final approval for publication. SKR helped with the study design and gave final approval for publication. All authors read and approved the final manuscript.
